# Pregnancy Outcomes in Three Distinct Uterine Anomalies: A Case Series

**DOI:** 10.7759/cureus.107820

**Published:** 2026-04-27

**Authors:** Megan M Woods, Allison K Oligschlaeger, Erica B Kingdon, Ashley L Alumbaugh

**Affiliations:** 1 Department of Medicine, A.T. Still University - Kirksville College of Osteopathic Medicine, Kirksville, USA; 2 Department of Obstetrics and Gynecology, Western Missouri Medical Center, Warrensburg, USA

**Keywords:** breech presentation, cesarean delivery, external cephalic version, malpresentation, müllerian anomalies, pregnancy outcomes, septate uterus, unicornuate uterus, uterine didelphys

## Abstract

This case series presents three patients with distinct Müllerian anomalies, highlighting variability in diagnosis, clinical management, and obstetric outcomes. Case 1 involved uterine didelphys with pregnancy in the right hemi-uterus, resulting in non-reassuring fetal heart rate tracings, prompting repeat cesarean delivery at 37 weeks' and six days' gestation. Case 2 involved a unicornuate uterus with a noncommunicating rudimentary horn and persistent breech presentation, requiring a scheduled cesarean delivery at 38 weeks' gestation. Case 3 involved a partial septate uterus associated with breech presentation and chronic hypertension, managed with cesarean delivery at 37 weeks' and three days' gestation. These cases highlight variability in clinical presentation and management across anomaly subtypes. All patients underwent individualized surveillance, including serial growth ultrasounds, cervical length monitoring, and antepartum testing, with favorable maternal and neonatal outcomes.

## Introduction

Müllerian anomalies arise from defects in duct formation, defects of fusion, or failure of resorption of the midline septum [1-3]. During embryogenesis, paired Müllerian ducts grow caudally and medially, fuse to form the uterovaginal canal, and subsequently reabsorb the intervening septum to create a single uterine cavity, cervix, and upper vagina [2,3]. Failure at any of these developmental stages results in distinct anatomic configurations: impaired duct formation leads to a hemi-uterus configuration, such as the unicornuate uterus; failure of duct fusion results in bicorporeal uteri, including bicornuate and didelphys; and incomplete resorption produces a septate uterus [1-3]. 

Although some Müllerian anomalies remain asymptomatic, their clinical importance lies in their association with adverse reproductive and obstetric outcomes. Women with uterine anomalies demonstrate higher rates of infertility, spontaneous miscarriage, fetal malpresentation, intrauterine growth restriction (IUGR), placental abruption, preterm delivery, and cesarean birth compared to women with normal uterine anatomy [3-6]. Obstetric risk varies by anomaly subtype, with a septate uterus conferring the highest risk of early pregnancy loss but also representing the most surgically correctable anomaly [1,4]. 

Uterine anomalies occur in about 5% of reproductive-age women, though true prevalence is likely underestimated due to asymptomatic presentation and delayed diagnosis [1,4,7]. Prevalence is substantially higher among individuals evaluated for infertility (approximately 8%) and among those with a history of pregnancy loss (approximately 13%), with the highest reported incidence occurring in patients experiencing both infertility and recurrent miscarriage (nearly 25%) [1]. In adolescents, Müllerian anomalies may present with primary amenorrhea, hematometrocolpos, or recurrent cyclic pelvic pain [1,2,8]. For adolescents presenting with these symptoms, pelvic ultrasound is typically the initial imaging modality due to its accessibility and cost-effectiveness, while MRI serves as the gold standard for detailed anatomical characterization when ultrasound findings are inconclusive or when surgical planning is required [4,8]. Obstructive variants typically present around menarche, whereas non-obstructive variants, such as a septate uterus, are more commonly diagnosed during infertility evaluation or following adverse pregnancy outcomes [1].

Importantly, Müllerian anomalies frequently coexist with congenital abnormalities of other organ systems due to shared embryologic origins. Renal anomalies, including unilateral renal agenesis, ectopic kidney, and collecting system abnormalities, occur in approximately 30-40% of patients with uterine anomalies, particularly in cases of unicornuate uterus and obstructed hemivagina and ipsilateral renal anomaly (OHVIRA) syndromes [4]. Skeletal abnormalities, such as scoliosis and vertebral segmentation defects, and less commonly cardiovascular anomalies have also been reported and may influence clinical presentation [2,9]. 

Müllerian anomalies are additionally associated with endometriosis, particularly in patients with obstructive variants. Retrograde menstruation is a common mechanism linking outflow obstruction to the development of endometriosis; however, endometriosis has also been reported in non-obstructive anomalies and in patients following surgical correction, suggesting a multifactorial pathogenesis [1,9]. This association is clinically relevant, as endometriosis may contribute to chronic pelvic pain, infertility, and adverse obstetric outcomes [1]. 

To address limitations of earlier classification systems, the American Society for Reproductive Medicine (ASRM) introduced the Müllerian Anomalies Classification (MAC) system in 2021. This clinically oriented, anatomy-based framework incorporates uterine, cervical, and vaginal anatomy while providing standardized definitions and preferred nomenclature [10]. Unlike the 1988 American Fertility Society classification, which lacked clear diagnostic criteria and excluded cervical and vaginal anomalies, the ASRM MAC improves diagnostic clarity, facilitates communication across disciplines, and supports consistent reporting of complex or combined variants [1,10]. 

Magnetic resonance imaging (MRI) is considered the gold standard for evaluating Müllerian anomalies due to its superior soft tissue contrast, multiplanar capability, and ability to delineate both the external uterine contour and the internal endometrial cavity [4,11]. Compared with ultrasonography or hysterosalpingography, MRI reliably differentiates anomalies of formation, fusion, and resorption and allows the comprehensive assessment of the pelvic anatomy, including the identification of associated renal anomalies and endometriosis [4,12]. Most importantly, MRI distinguishes septate uteri, characterized by a normal or mildly indented fundal contour and amenable to hysteroscopic resection, from bicorporeal anomalies with a deep external fundal cleft, which are not surgically correctable [4]. 

Despite recognition of increased reproductive and obstetric risk, comparative outcome data across specific Müllerian anomaly subtypes remain limited, particularly within a single institution. Meta-analyses demonstrate increased odds of IUGR and placental abruption among patients with uterine anomalies; however, variability in study design and classification systems limits direct comparison across anomaly types [5,6]. 

The purpose of this case series is to describe the pregnancy course, delivery characteristics, and obstetric outcomes in three patients with distinct Müllerian anomalies, highlighting variability in clinical presentation and pregnancy risk across anomaly subtypes.

## Case presentation

Case 1: uterine didelphys

**Figure 1:**
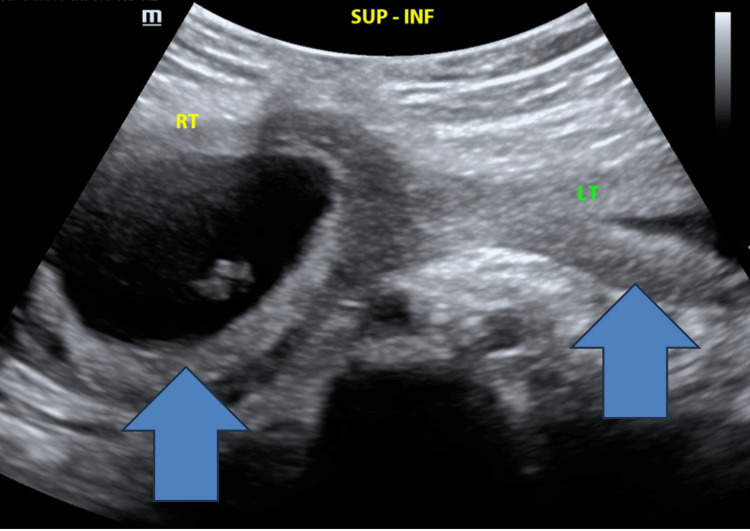
Transabdominal pelvic ultrasound at eight weeks' and five days' gestation demonstrating uterine didelphys. A gestational sac with yolk sac is visualized within the right hemi-uterus. A second, separate uterine cavity consistent with the left hemi-uterus is also visualized without evidence of pregnancy. Arrows indicate the gestational sac within the right hemi-uterus and the separate left hemi-uterus. These findings are consistent with uterine didelphys (original clinic image).

A 23-year-old gravida 6, para 1 (G6P1041: 1 term, 0 preterm, 4 abortions, 1 living) woman with a known diagnosis of uterine didelphys presented for delivery at 37 weeks' and six days' gestation. Her anomaly consisted of two fully developed hemi-uteri with separate endometrial cavities, a duplicated cervix, and a longitudinal vaginal septum. The diagnosis had been established on prior obstetric imaging. Her obstetric history was significant for four first-trimester spontaneous abortions and one prior term cesarean delivery for breech presentation, with that pregnancy occurring in the left uterus. Early ultrasound findings consistent with uterine didelphys are shown in Figure 1.

The current pregnancy was located in the right uterus. The patient received progesterone supplementation due to a history of recurrent pregnancy loss, per prior clinical management. Prenatal care included maternal-fetal medicine (MFM) consultation, serial growth ultrasounds, and cervical length monitoring, all of which remained reassuring. No antenatal complications such as growth restriction, malpresentation, hypertensive disorders, or abnormal amniotic fluid volume were identified. 

The patient desired a trial of labor after cesarean (TOLAC). She presented in spontaneous labor with cephalic fetal presentation; however, labor was complicated by non-reassuring fetal heart rate tracings, prompting repeat cesarean delivery. A viable female neonate weighing 6 lb 9 oz was delivered with Apgar scores of 9 and 9. The neonatal course was uncomplicated aside from mild hyperbilirubinemia related to ABO incompatibility, which was managed conservatively. The maternal postoperative course was uncomplicated.

Case 2: unicornuate uterus with noncommunicating rudimentary horn

**Figure 2:**
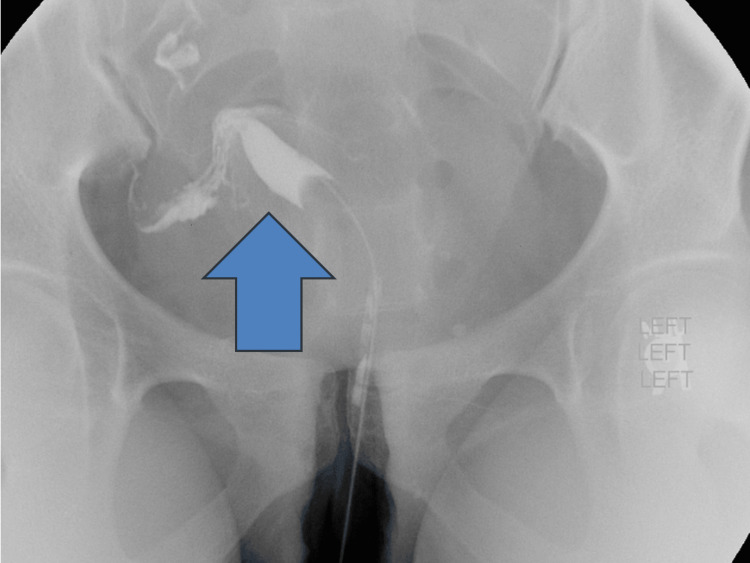
Hysterosalpingogram performed for infertility evaluation demonstrating a unicornuate uterus. Contrast opacifies a single asymmetric uterine cavity with unilateral tubal filling. No opacification of a contralateral uterine cavity is visualized, supporting the diagnosis of a unicornuate uterus with a noncommunicating rudimentary horn. Arrow indicates the single asymmetric uterine cavity (original clinic image).

A 31-year-old primigravida (G1P0) woman with a history of polycystic ovary syndrome, insulin resistance, and right-sided salpingitis isthmica nodosa conceived via in vitro fertilization. Her uterine anomaly, a unicornuate uterus with a normally developed right hemi-uterus, a normal right fallopian tube and ovary, and a noncommunicating rudimentary left horn with an atretic fallopian tube, had been diagnosed prior to pregnancy using hysterosalpingography (Figure 2).

The patient had a history of infertility.

The pregnancy was monitored closely by MFM with serial growth ultrasounds, cervical length surveillance, and antenatal testing beginning at 32 weeks due to chronic decreased fetal movement. Cervical length remained normal throughout pregnancy. The fetus demonstrated persistent breech presentation. External cephalic version (ECV) was deferred due to the known uterine anomaly and anticipated low likelihood of success.

The pregnancy was further complicated by preeclampsia without severe features. At 38 weeks' gestation, the patient underwent a scheduled primary cesarean delivery. A male neonate weighing 8 lb 5 oz was delivered with Apgar scores of 8 and 8. The neonatal course was notable for transient respiratory distress requiring brief noninvasive respiratory support and hyperbilirubinemia, both of which resolved before discharge. Postpartum, the patient received magnesium sulfate for seizure prophylaxis and was continued on antihypertensive therapy with good blood pressure control. 

Case 3: suspected partial septate uterus

A 27-year-old gravida 4, para 1 (G4P1021) woman presented for delivery at 37 weeks' and three days' gestation. Her obstetric history was notable for one prior term delivery complicated by preeclampsia requiring magnesium sulfate, followed by two first-trimester spontaneous abortions at five and six weeks' gestation. Her medical history included chronic hypertension, class III obesity (BMI 40), polycystic ovary syndrome, anxiety, depression treated with sertraline, and insulin resistance managed with metformin. The current pregnancy was complicated by chronic hypertension with worsening blood pressure control, first-trimester bleeding requiring progesterone supplementation, and polyhydramnios. 

Prenatal care included MFM consultation with serial growth ultrasounds and antenatal surveillance. The fetus demonstrated persistent breech presentation. An ECV was attempted at term for persistent malpresentation following routine counseling regarding risks, benefits, and likelihood of success, in accordance with the standard of obstetric practice.

During cesarean delivery, a previously unrecognized uterine anomaly suspicious for a partial septate uterus was identified intraoperatively. A viable male neonate was delivered with Apgar scores of 6 and 8 at one and five minutes, respectively, and a birth weight of 6 lb 4 oz. Prenatal ultrasound had demonstrated mild renal pelvic dilation; however, postnatal renal ultrasound was unremarkable. The neonate was discharged on postpartum day 3 and was briefly readmitted for hyperbilirubinemia. The maternal postoperative course was otherwise uncomplicated.

## Discussion

**Figure 3:**
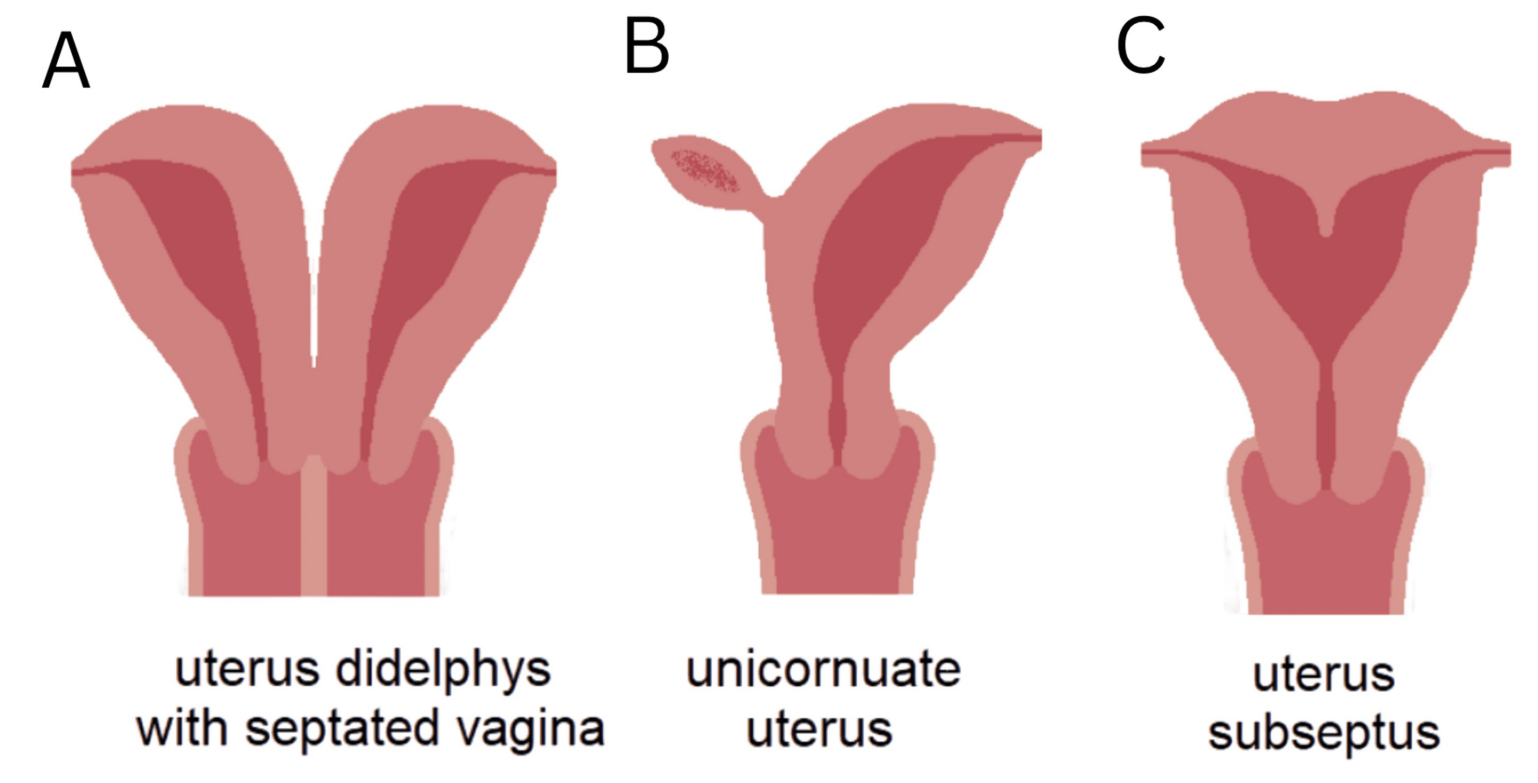
Representative uterine anomaly subtypes. (A) Uterine didelphys: complete duplication of uterine cavities, two cervices, and +/- vaginal septum. (B) Unicornuate uterus: single hemi-uterus with noncommunicating rudimentary horn. (C) Partial septate uterus: normal external fundal contour, internal septum diving endometrial cavity/extending partially into cavity. Figure created by the authors using Canva; adapted from [13] under the Creative Commons CC0 1.0 Universal Public Domain Dedication

This case series highlights obstetric outcomes across distinct Müllerian anomaly subtypes and underscores the importance of accurate anatomic classification and the timing of diagnosis in pregnancy management. Although all three patients achieved favorable maternal and neonatal outcomes, their pregnancy courses and delivery decisions differed based on uterine anatomy, associated obstetric risks, and whether the anomaly was known before delivery. Representative Müllerian anomaly subtypes are illustrated in Figure 3.

Malpresentation emerged as the most consistent complication across cases, occurring in both the unicornuate and suspected partial septate uterus and contributing directly to cesarean delivery. This finding aligns with existing literature demonstrating elevated rates of breech presentation in patients with unicornuate and septate uteri due to asymmetric or constrained uterine cavities that limit fetal mobility [3,5]. In patients with uterine didelphys, malpresentation was present in prior pregnancy but not in the most recent pregnancy, illustrating the variability in presentation even within the same anomaly subtype and between hemi-uteri. A comparison of clinical features and obstetric outcomes across cases is summarized in Table 1.

**Table 1:** Comparative obstetric outcomes by uterine anomaly. TOLAC: trial of labor after cesarean; ECV: external cephalic version

Uterine anomaly	Key feature influencing delivery decision	Diagnosis timing	Management strategy	Delivery outcome
Uterine didelphys	Desired TOLAC --> fetal intolerance of labor	Known antenatally	Trial of labor with continuous monitoring	Repeat cesarean at 37w6d
Unicornuate uterus with noncommunicating rudimentary horn	Persistent breech presentation	Known antenatally	Planned cesarean delivery	Cesarean at 38w
Partial septate uterus	Breech presentation; unsuccessful ECV prior to diagnosis	Suspected intraoperatively	Attempted ECV, cesarean for persistent breech	Cesarean at 37w3d

Diagnosis timing significantly influenced clinical decision-making. In the unicornuate uterus case, antenatal recognition of the anomaly influenced the decision to defer ECV, consistent with recommendations that consider uterine anomalies a relative contraindication due to reduced success rates and theoretical risks [5]. In contrast, the partial septate uterus was only suspected intraoperatively, and ECV was attempted before the recognition of abnormal uterine anatomy. This distinction emphasizes the clinical consequences of delayed or missed diagnosis, particularly when management decisions such as mode of delivery or eligibility for version are being considered. 

The uterine didelphys case further illustrates how known anatomy can support individualized delivery planning. Despite the presence of a congenital uterine anomaly and prior cesarean delivery, a TOLAC was pursued due to reassuring antenatal findings and cephalic presentation. Although labor was ultimately complicated by non-reassuring fetal heart rate tracings, the decision to attempt TOLAC was informed by the known anomaly subtype and close intrapartum monitoring, reinforcing that Müllerian anomalies alone do not universally prevent vaginal delivery attempts [1,4]. 

Consistent with prior studies, obstetric outcomes in this series varied by anomaly subtype but were generally favorable with tailored surveillance [5,6]. All patients received MFM consultation, serial growth ultrasounds, cervical length monitoring, and antenatal testing when clinically indicated. Importantly, no cases were complicated by fetal growth restriction or preterm birth, outcomes that have been reported at higher rates in larger cohorts of patients with uterine anomalies [5,6]. These findings suggest that early identification and individualized management may mitigate some of the risks traditionally associated with Müllerian anomalies. A limitation of this report is that all the patients were around the same age and presented with symptoms in adulthood and during pregnancy. Presentation of anomalies in adolescents was not measured in this report, but should be documented in future case studies to help determine long-term management and possible reproductive involvement in these patients. 

Accurate classification is critical, particularly in differentiating septate uteri from bicorporeal anomalies, as management and long-term reproductive implications differ substantially [10]. MRI remains the gold standard for definitive diagnosis, especially when ultrasound findings are unclear or when diagnosis may alter obstetric or surgical planning [4,11,12]. Broader adoption of standardized classification systems, such as the ASRM MAC, may improve consistency in diagnosis, reporting, and counseling across institutions [10].

## Conclusions

Müllerian anomalies are associated with variable pregnancy risks that differ by anomaly subtype and are strongly influenced by the timing and accuracy of diagnosis. This case series demonstrates that malpresentation and intolerance of labor are common clinical challenges, while favorable maternal and neonatal outcomes are achievable with individualized surveillance and delivery planning. Early and accurate identification of uterine anomaly subtype supports informed counseling, appropriate use of interventions such as ECV or trial of labor, and optimized perinatal care. Further comparative research across anomaly subtypes is needed to better define outcome patterns and guide evidence-based management strategies.
